# German pediatric intensive care transport registry: study protocol for a prospective multicenter registry

**DOI:** 10.3389/fped.2025.1669094

**Published:** 2025-10-07

**Authors:** Stefan Winkler, Felix Dittgen, Edmondo Hammond, Nele Börner, Johanna Kossack, Frank Eifinger, Ingeborg Alijda van den Heuvel, Elias Klinghammer, Victoria Lieftüchter, Pia Paul, Alba Perez-Ortiz, Patricia Bimboese, Richard Biedermann, André Jakob, Sarah Irlbeck, Nadine Mand

**Affiliations:** ^1^ Division of Pediatric Intensive Care Medicine, Department of Pediatrics, University Hospital Carl Gustav Carus, Technische Universität Dresden, Dresden, Germany; ^2^ Department of Neonatology and Pediatric Intensive Care Medicine, Universitätsmedizin Mannheim, Mannheim, Germany; ^3^ Department of Pediatrics, Diakonie Kliniken Bad Kreuznach gGmbH, Bad Kreuznach, Germany; ^4^ Department of Pediatric Respiratory Medicine, Immunology and Critical Care Medicine, Charité University Hospital Berlin, Berlin, Germany; ^5^ Division of Neonatology and Pediatric Intensive Care, Department of Pediatrics, Faculty of Medicine and University Hospital Cologne, University of Cologne, Cologne, Germany; ^6^ Department of General Pediatrics, University Hospital Münster, Münster, Germany; ^7^ Neonatology and Pediatric Intensive Care Medicine, Department of Pediatrics, Philipps-University Marburg, Marburg, Germany; ^8^ Pediatric Intensive Care Medicine, Dr. v. Hauner Children’s Hospital, LMU University Hospital, LMU Munich, Munich, Germany; ^9^ Department of Pediatrics, University Hospital of Würzburg, Würzburg, Germany; ^10^ Department of Pediatrics, University Hospital Jena, Jena, Germany; ^11^ Department of Pediatrics, Interdisciplinary Pediatric Intensive Care Medicine, University Medical Center Rostock, Rostock, Germany; ^12^ Pediatric Intensive Care Unit, Children’s Hospital and Department of Anesthesiology and Intensive Care Medicine, Medical Faculty, Otto-von-Guericke-University Magdeburg, Magdeburg, Germany

**Keywords:** pediatric, interhospital transfer, critical care, intensive care, transport, emergency medical service, registry

## Abstract

**Background:**

In contrast to critical care transport of adults or newborns, transport of pediatric critical care patients in Germany is neither regulated by law nor centrally organized. Due to their different therapeutic needs compared to newborns or adults, critically ill children may receive insufficient treatment during transport. In some regions in Germany, pediatric centers provide specialized pediatric retrieval teams, while others organize each transport individually. Currently, no valid data on pediatric critical care transports in Germany are available, nor are they recorded in a structured manner.

**Objectives:**

To establish a nationwide registry for pediatric intensive care transports in Germany. The aim is to describe and analyze the need for and current practice of specialized transports. This data may be used for future demand planning.

**Setting:**

Transports are documented by pediatric centers admitting pediatric patients via intensive care transports.

**Inclusion criteria:**

All interhospital pediatric intensive care transports of children, aged >27 days and >41 + 0 weeks of corrected gestational, age to <18 years, are eligible for data entry.

**Methods:**

The study is designed as a prospective, multicenter registry. Transport data will be collected locally at participating pediatric centers and then submitted digitally and anonymized via a secure, web-based platform.

**Discussion:**

We anticipate high participation from pediatric intensive care units and expect to present valid data on the need for pediatric intensive care transports in Germany. This data may serve as a foundation for nationwide demand planning for pediatric intensive care transport resources.

## 1 Introduction

While pediatric services in Germany lack the centralized structure seen in the United Kingdom or the United States, pediatric intensive care is mainly concentrated in large pediatric centers. Critically ill infants and children are typically transported to the nearest children's hospital, where their condition is assessed, initial stabilization is provided, and transfer to a pediatric intensive care unit (PICU) is coordinated. Referral of critically ill children to these centers or PICUs necessitates specialized critical care transport. In retrospective association studies, transport by specialist pediatric critical care transport teams (PCCTs) was associated with fewer transport-related adverse events or interventions needed directly after admission (1, 2). Non-specialist transport teams often reported feeling uncomfortable or uncertain when administering pediatric medication or trauma care prehospitally (3–6), thus negatively influencing parents’ experience of their child's emergency transfer (7). Prospective cohort studies demonstrated a significant improvement in survival rates for patients transferred by PCCTs, after adjustment for illness severity (8, 9). However, establishing and maintaining PCCTs requires significant resources (10). Currently, unlike adult or neonatal critical care transport, pediatric critical care transport in Germany is neither legally regulated nor centrally organized. Consequently, retrieval of critically ill children is often performed by general emergency medical services (EMS) or adult critical care transport teams, with occasional support from pediatric specialists. These non-specialized teams are frequently available but may have limited pediatric training and experience in managing true pediatric emergencies. Furthermore, their equipment may be inappropriate for optimal care of pediatric patients. In contrast to Germany, numerous countries run nationwide or large-scale PCCT programs (11, 12). In the United Kingdom, pediatric intensive care services were centralized over 20 years ago. Subsequently, PCCTs were set up for each region to transport critically ill children from acute general hospitals to dedicated PICUs (13, 14). Transport cases account for almost half of the 12,000 annual emergency admissions to UK PICUs, with the majority of these cases handled by PCCTs (15).

To date, no published nationwide data exist on pediatric critical care transports in Germany. To address these challenges, we initiated the development of a nationwide pediatric critical care transport registry. This report outlines the rationale, framework, development, and refinement of the German “Pediatric Intensive Care Transport Registry” (PIT Registry) dataset, as well as the registry's operational methods.

## 2 Methods and analysis

### 2.1 Goals of the registry

The Pediatric Intensive Care Transport Registry is designed as a prospective multi-center registry. It was initiated to establish a research infrastructure for the systematic documentation of pediatric intensive care transports across Germany. The PIT registry aims to improve the quality and accessibility of care for critically ill children by providing a comprehensive dataset covering transport characteristics, patient demographics, medical interventions, clinical status upon admission, and transport-related adverse events.

The primary objective of the PIT registry is to collect timely, nationwide data on pediatric intensive care transports to address relevant clinical and research questions. The secondary aim is to assess the actual demand for pediatric intensive care transport services in Germany and to compare these needs with existing transport capacities and organizational structures.

### 2.2 Governance

The registry was formally initiated in June 2023 by the Transport Working Group of the Pediatric Section of the German Interdisciplinary Association for Intensive Care and Emergency Medicine (DIVI) and has since received official endorsement from the DIVI. The registry was launched in November 2024, with the first patient being enrolled on 25 November 2024.

Its coordination is overseen by a steering committee comprising four members representing three university medical faculties (Dresden, Mannheim, Marburg) and one regional pediatric center (Bad Kreuznach). In addition to the formal management of the registry, the steering committee is also responsible for public relations and communication management, onboarding and training of newly participating centers, as well as data management. Virtual steering committee meetings are held biweekly to support this process.

As the PIT registry is not based on an existing network of children's hospitals, a system of regional PIT coordinators was implemented across the German federal states, with one or two coordinators assigned to each state. These coordinators are responsible for the decentralized recruitment of local PICUs and for obtaining the necessary approvals from local ethics committees. They also form the Scientific Advisory Board. Virtual meetings with the coordinators are held every month.

All German hospitals with pediatric intensive care units that receive pediatric critical care transports are eligible to participate in the PIT registry, regardless of patient volume. Each participating pediatric center has a designated local representative (PIT investigator) who ensures on-site data collection and serves as the registry's contact person. New participating centers receive training from members of the steering committee on the study population and data entry procedures, thereby promoting data completeness and comprehensiveness. These trainings are offered on a weekly basis. Ongoing communication with local sites is ensured through dedicated email contacts.

The steering committee, regional coordinators, and local investigators of the Pediatric Intensive Care Transport Registry collectively form the PIT study group.

### 2.3 Study population

All interhospital pediatric intensive care transports of children, aged >27 days and >41 + 0 weeks of corrected gestational age, to <18 years, are eligible for data entry.

**Figure 1:**
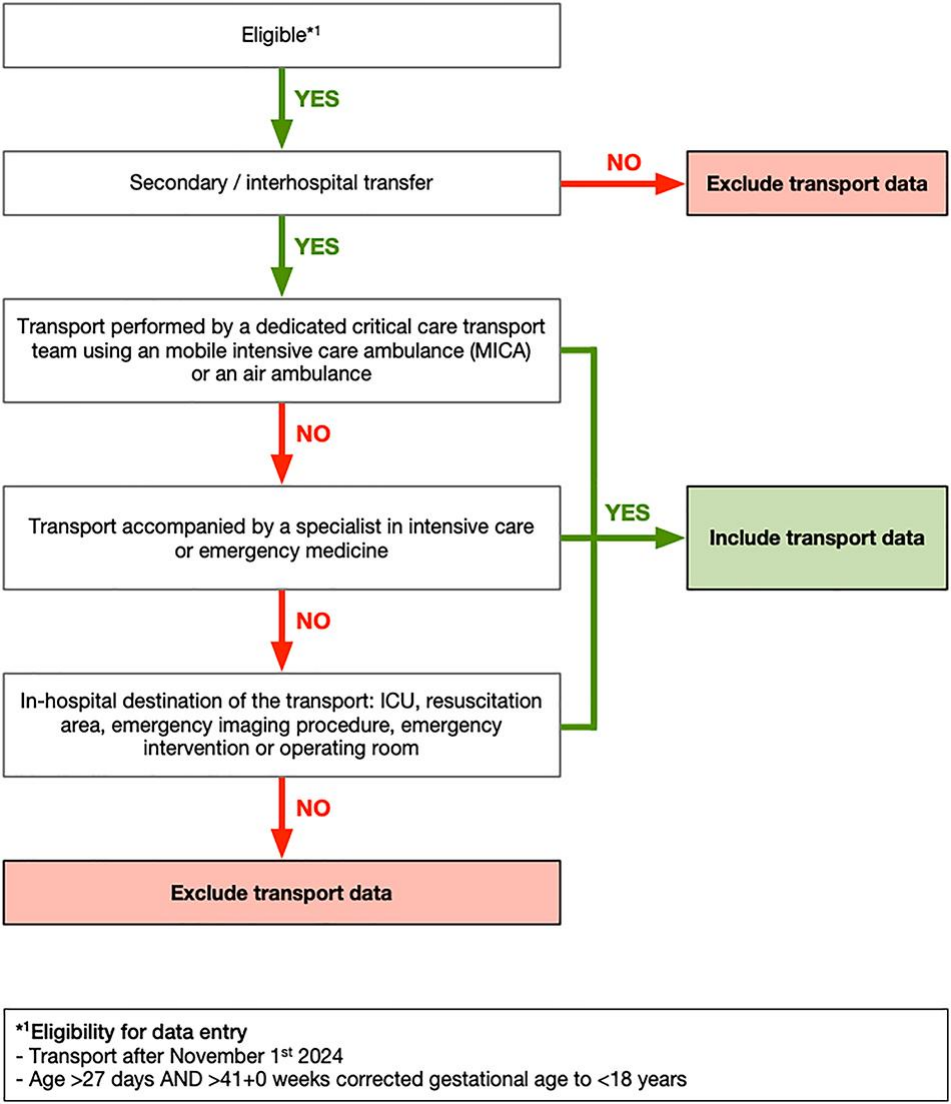
Inclusion criteria for transport datasets. ICU, intensive care unit.

Critical care transports are defined as transports that meet at least one of the following criteria: I) The transport was performed by a dedicated critical care transport team using a mobile intensive care ambulance (MICA) or an air ambulance. II) The transport was accompanied by a specialist in intensive care or emergency medicine. III) The in-hospital destination of the transport is a critical care area, such as an intensive care unit, a resuscitation area, an operating room, an emergency imaging procedure, or an emergency intervention (see Figure 1).

### 2.4 Dataset

The registry dataset was developed by experts in the field of pediatric intensive care and pediatric critical care transport through a two-stage Delphi process (16). It consists of 71 variables in four categories: (I) Details on transport timing, referral, and accepting hospitals; (II) Patient details including vitals on arrival; (III) Details on the transport team and the transport equipment; (IV) Interventions by the transport teams and critical incidents. The minimum dataset comprises seven mandatory variables (date of transport, collection unit, collection area, destination unit, destination type, patient's age, mode of transport). Both English and German versions of the data dictionary can be found on the PIT registry homepage (https://www.pit-register.de), Mendeley Data (DOI: 10.17632/nngrkzxjf8.1) and in the supplement (see Supplementary Tables S1, S2).

The dataset will be evaluated for completeness and plausibility on a regular basis. After the first six months of data collection, local investigators from the participating pediatric intensive care units (PICUs) will be consulted on potential improvements to the collected variables. Based on this feedback, selected variables will be revised as necessary. To ensure the registry is continuously refined, local PICU investigators will regularly be involved in the review process. All modifications to the set of variables will be systematically documented to maintain the highest level of transparency.

### 2.5 Data management

To ensure comprehensive documentation of pediatric critical care transports and to prevent duplicate records resulting from multiple entries, transports are documented by pediatric centers that admit patients via critical care transport. Upon admission, eligible patients’ transport data is documented using a standardized paper-and-pencil sheet. Secondary, local PIT investigators enter fully anonymized patient data via electronic case report forms (eCRFs) into REDCap (Research Electronic Data Capture) (17, 18). This Data is stored on a server at the Coordinating Centre for Clinical Trials (CCC) of Philipps-University Marburg (Germany).

Patient data are fully anonymized so that reported transports cannot be re-identified by CCC personnel or scientists analyzing the data. The study and data protection protocol were initially approved by the ethics committees at the Technical University Dresden (Germany) (study ID: REG-EK-270072024). Subsequently, the study protocol was approved at the local ethics committees of all participating pediatric centers.

All members of the PIT study group are eligible to apply to use PIT registry data to answer specific scientific questions. Study proposals can be submitted via the Use and Access Committee (UAC), which comprises rotating members of the Steering Committee and the Scientific Advisory Board. Further details have been published in the dedicated publication guidelines on the PIT registry website.

### 2.6 Data monitoring and data quality

Routine data quality checks will be conducted at regular intervals, especially during the initial period of the registry, to ensure accuracy and consistency. Each PIT investigator is responsible for ensuring the correctness and plausibility of their data before submitting it. However, as the registry is fully anonymized, individual centers will not be able to modify data once it has been submitted.

As data will be checked for completeness and plausibility, single centers will be audited and re-trained when a high percentage of missing or implausible values appear. Items with a high proportion of missing values are regularly re-evaluated and adjusted if necessary.

The quality of the survey methods is investigated on an annual basis. Quality indicators comprise (I) the nationwide coverage of the PIT registry (based on eligible and participating centers), (II) the completeness of values per case and possible reasons for incompleteness, and (III) the amount of and reasons for implausible values.

The registry data are evaluated annually by the steering committee, in collaboration with statisticians and epidemiologists. The resulting annual reports are distributed to all participating centers by the end of the first quarter of each year.

As the registry is planned as a long-term, open-ended registry, it is intended to evolve and change with emerging research questions.

### 2.7 Statistical analysis

Statistical analyses are performed continuously for monitoring purposes. Patient demographics and basic transport variables will be summarized using descriptive summary measures. Further analysis will be performed according to specific research questions and will be undertaken by a trained statistician or epidemiologist. Upon request, only evaluated data will be provided for study purposes, not original data.

## 3 Discussion

The Pediatric Intensive Care Transport Registry aims to collect transport data on a nationwide basis, providing the first comprehensive and reliable data on pediatric critical care transports across Germany.

As EMS in Germany are regulated by the federal states on a regional basis, the result is a heterogeneous landscape of transport systems for patients of all ages. Regional studies focusing on EMS for pediatric-out-of hospital emergencies indicate a low incidence of critical illness among children (19) and a high rate of inappropriate utilization for mildly ill pediatric cases (20). Local infrastructure for the interhospital transfer of critically ill children is limited to individual regional projects (21) and there is a lack of nationwide systems dedicated to their transport. This poses significant challenges for the systematic collection and analysis of relevant data, which are to be addressed through the PIT registry.

Clinical registries have become a vital tool for performance measurement, quality improvement, and clinical research. They expose variations in practices, processes, and outcomes, and identify targets for improvement (22). In pediatrics, registries have been associated with improved outcomes, particularly in the management of preterm neonates, in pediatric oncology, pediatric critical care, and in critical care transport (9, 23–28). Recently, a nationwide pediatric registry focusing on pediatric intensive care admissions (PIA) in Germany has been initiated (29). This network aims to improve pediatric intensive care medicine in Germany by providing a comprehensive understanding of critical illness, benchmarking treatment quality, and enabling disease surveillance. Integrating data from the PIT registry with the PIA network may enhance future analysis of outcomes in critically ill children affected by intensive care transport.

The systematic collection of data on pediatric critical care transports within the PIT registry facilitates the identification of current practices and needs. In the future, these data will support needs-based planning, the development of quality indicators, and the design of targeted training programs for the personnel involved in pediatric intensive care transports.

The registry has several limitations: (I) Due to the lack of standardized software interfaces for patient management data systems (PDMS) that integrate with the study database, the data entry process is currently manual. This additional burden on already limited human resources in German hospitals may compromise the likelihood of complete data entry. To ensure the completeness of data, routine data quality checks are conducted, and centers will be re-trained when a high percentage of missing or implausible values appear. (II) Since registry participation is voluntary, a comprehensive database encompassing all pediatric critical care transports performed in Germany seems unlikely to be achieved. Nevertheless, the decentralized structure based on regional study coordinators assigned to the German federal states has proven highly effective in inviting regional pediatric centers for study participation and supporting them with applications to their local Institutional Review Boards. (III) To avoid interfering with already established neonatal registries and to ensure comparability with the population represented in the German PIA network, only non-neonatal cases are included in the registry. However, this approach may result in the omission of some transports conducted by pediatric critical care teams. As the registry is intended to evolve, a future adjustment of the inclusion criteria is conceivable. (IV) Local PIT investigators are responsible for data entry, and no monitoring is available to ensure the completeness and quality of the captured data. Therefore, regional study coordinators will play a key role in maintaining close interaction with participating centers to encourage their active participation in the study. (V) No government or institutional funding is currently available to support the project, which poses a significant risk to its long-term continuation.

To overcome these limitations and to unlock its full potential, the registry will require ongoing refinement and advancement of its methods. The optimized digitalization of hospital documentation and automated data export to study databases are essential in minimizing the workload of personnel involved and facilitating the collection of high-quality data. Close interaction between regional study coordinators and participating centers, as well as transparent rules for data availability, which is granted to all members of the PIT study group, are key elements for active study participation.

In summary, the PIT registry is a nationwide pediatric critical care transport registry that aims to improve the availability and quality of transports of critically ill children across Germany. Regional study coordinators play a key role in encouraging pediatric centers for active participation. In the future, potential collaborative data collection between the PIA network and the PIT registry may further enhance the possibilities for analysis and improve the reliability of the data.

## References

[1] Vos GD Nissen AC Nieman HM Meurs F Waardenburg MMB van DA Comparison of interhospital pediatric intensive care transport accompanied by a referring specialist or a specialist retrieval team. Intensiv Care Med. (2004) 30:302–8. 10.1007/s00134-003-2066-7 14618230

[2] Prabhudesai S Kasala M Manwani N Krupanandan R Ramachandran B. Transport-related adverse events in critically-ill children: the role of a dedicated transport team. Indian Pediatr. (2017) 54:942–5. 10.1007/s13312-017-1187-y 28849766

[3] Tsao HS Sutcliffe T Wang C Vargas SE Day C Brown LL. Barriers and enablers in prehospital pediatric analgesia. Prehospital Emerg Care. (2024) 29:1–7. 10.1080/10903127.2024.2431586 39561318

[4] Carey JM Studnek JR Browne LR Ostermayer DG Grawey T Schroter S Paramedic-identified enablers of and barriers to pediatric seizure management: a multicenter, qualitative study. Prehospital Emerg Care. (2019) 23:870–81. 10.1080/10903127.2019.1595234 30917730

[5] Shah MI Ostermayer DG Browne LR Studnek JR Carey JM Stanford C Multicenter evaluation of prehospital seizure management in children. Prehospital Emerg Care. (2021) 25:475–86. 10.1080/10903127.2020.1788194 32589502

[6] Patel SC Murphy S Penfil S Romeo D Hertzog JH. Impact of interfacility transport method and specialty teams on outcomes of pediatric trauma patients. Pediatr Emerg Care. (2018) 34:467–72. 10.1097/pec.0000000000001167 28463947

[7] Evans REC Barber V Ramnarayan P Davies P Wray J, Group on behalf of the DS. Emergency inter-hospital transfer of children to PICUs in the United Kingdom: qualitative exploration of Parents’ experiences of retrieval teams. Pediatr Crit Care Med. (2023) 24:e476–86. 10.1097/pcc.0000000000003267 37166250

[8] Orr RA Felmet KA Han Y McCloskey KA Dragotta MA Bills DM Pediatric specialized transport teams are associated with improved outcomes. Pediatrics. (2009) 124:40–8. 10.1542/peds.2008-0515 19564281

[9] Ramnarayan P Thiru K Parslow RC Harrison DA Draper ES Rowan KM. Effect of specialist retrieval teams on outcomes in children admitted to paediatric intensive care units in England and Wales: a retrospective cohort study. Lancet. (2010) 376:698–704. 10.1016/s0140-6736(10)61113-0 20708255

[10] Kamidani R Okada H. Centralization and transport of critically ill pediatric patients. Front Pediatr. (2025) 13:1601875. 10.3389/fped.2025.1601875 40535697 PMC12174163

[11] Ramnarayan P Wood D Draper E Palmer L Feltbower R Buckley HL Transport of critically ill children to paediatric intensive care units in the UK and Ireland: 2013–2022. Arch Dis Child. (2025) 110:127–32. 10.1136/archdischild-2024-327088 39209528

[12] Abdel-Latif ME Berry A. Analysis of the retrieval times of a centralised transport service, New South Wales, Australia. Arch Dis Child. (2009) 94:282. 10.1136/adc.2007.125211 18927147

[13] Health D of. Paediatric Intensive Care: A Framework for the Future. Wetherby: United Kingdom, Department of Health (1997).

[14] White M Weir PM Garland L Edees S Henderson AJ. Outcome of critically ill children before and after the establishment of a pediatric retrieval service as a component of a national strategy for pediatric intensive care. Pediatr Crit Care Med. (2002) 3:255–60. 10.1097/00130478-200207000-00010 12780966

[15] Network PIC. National Report of the Paediatric Intensive Care Audit Network, January 2011 - December 2013. Leeds, United Kingdom: Universities of Leeds and Leicester (2014).

[16] Niederberger M Deckert S. Das Delphi-verfahren: methodik, varianten und anwendungsbeispiele. Z Evid Fortbild Qual Gesundhwes. (2022) 174:11–9. 10.1016/j.zefq.2022.08.007 36137932

[17] Harris PA Taylor R Thielke R Payne J Gonzalez N Conde JG. Research electronic data capture (REDCap)—a metadata-driven methodology and workflow process for providing translational research informatics support. J Biomed Inform. (2009) 42:377–81. 10.1016/j.jbi.2008.08.010 18929686 PMC2700030

[18] Harris PA Taylor R Minor BL Elliott V Fernandez M O’Neal L The REDCap consortium: building an international community of software platform partners. J Biomed Inform. (2019) 95:103208. 10.1016/j.jbi.2019.103208 31078660 PMC7254481

[19] Eich C Russo SG Heuer JF Timmermann A Gentkow U Quintel M Characteristics of out-of-hospital paediatric emergencies attended by ambulance- and helicopter-based emergency physicians. Resuscitation. (2009) 80:888–92. 10.1016/j.resuscitation.2009.05.008 19520484

[20] Poryo M Burger M Wagenpfeil S Ziegler B Sauer H Flotats-Bastardas M Assessment of inadequate use of pediatric emergency medical transport services: the pediatric emergency and ambulance critical evaluation (PEACE) study. Front Pediatr. (2019) 7:442. 10.3389/fped.2019.00442 31709211 PMC6823188

[21] Winkler S Schawohl A Waurig F Busch D Beer A Galow L Verbesserung der flächendeckenden versorgung kritisch kranker kinder durch telemedizin und pädiatrische intensivtransporte: das kinder tele-intensivnetzwerk sachsen (KIdS). Monatsschr Kinderheilkd. (2025) 173:856–66. 10.1007/s00112-025-02263-0

[22] Nelson EC Dixon-Woods M Batalden PB Homa K Citters ADV Morgan TS Patient focused registries can improve health, care, and science. Br Med J. (2016) 354:i3319. 10.1136/bmj.i3319 27370543 PMC5367618

[23] Gale C Morris I Chivers Z Costeloe K Dopran J Dorling J The UK national neonatal research database: using neonatal data for research, quality improvement and more. Arch Dis Child - Educ Pr Ed. (2016) 101:216. 10.1136/archdischild-2015-309928 PMC4975807 26968617

[24] Humberg A Fortmann MI Spiegler J Rausch TK Siller B Silwedel C Recurrent late-onset sepsis in extremely low birth weight infants is associated with motor deficits in early school age. Neonatology. (2022) 119:695–702. 10.1159/000525709 36327925

[25] Schrappe M Reiter A Zimmermann M Harbott J Ludwig W-D Henze G Long-term results of four consecutive trials in childhood ALL performed by the ALL-BFM study group from 1981 to 1995. Leukemia. (2000) 14:2205–22. 10.1038/sj.leu.2401973 11187912

[26] Heneghan JA Akande MY Ramgopal S Evans MD Hallman M Goodman DM. New morbidities during critical illness and associated risk of ICU readmission: virtual pediatric systems cohort, 2017–2020. Pediatr Crit Care Med. (2024) 25:e405–9. 10.1097/pcc.0000000000003542 38780383 PMC11449647

[27] Namachivayam SP Carlin JB Millar J Alexander J Edmunds S Ganeshalingham A Gestational age and risk of mortality in term-born critically ill neonates admitted to PICUs in Australia and New Zealand. Crit Care Med. (2020) 48:e648–56. 10.1097/ccm.0000000000004409 32697505

[28] Ramnarayan P Dimitriades K Freeburn L Kashyap A Dixon M Barry PW Interhospital transport of critically ill children to PICUs in the United Kingdom and republic of Ireland. Pediatr Crit Care Med. (2018) 19:e300–11. 10.1097/pcc.0000000000001506 29432405

[29] Bruns N Dohna-Schwake C Olivieri M Urschitz MS Blomenkamp S Frosch C Pediatric intensive care unit admissions network—rationale, framework and method of operation of a nationwide collaborative pediatric intensive care research network in Germany. Front Pediatr. (2024) 11:1254935. 10.3389/fped.2023.1254935 38269291 PMC10806156

