# Supplementary material for: German pediatric intensive care transport registry: study protocol for a prospective multicenter registry
Source: Front Pediatr. 2025 Oct 7;13:1669094. doi: 10.3389/fped.2025.1669094 (PMC12537787; doi:10.3389/fped.2025.1669094)
Supplement: Supplementary file 1 [file Datasheet1.pdf]

frontiers

Supplementary Material

German Pediatric Intensive Care Transport Registry (PIT): Study Protocol for a Prospective Multicenter Registry

Stefan Winkler, Felix Dittgen, Edmondo Hammond, Nele Börner, Johanna Kossack, Frank Eifinger, Ingeborg Alijda van den Heuvel, Elias Klinghammer, Victoria Lieftüchter, Pia Paul, Alba Perez-Ortiz, Patricia Bimboese, Richard Biedermann, André Jakob, Sarah Irlbeck, Nadine Mand

1 Supplementary Tables

Supplementary Table 1: Pediatric Intensive Care Transport Registry (PIT) - Data Dictionary / Item List V1.0 – *English version*

#	Variable / Field Label	Field Type	Field Attributes
	**Basic transport information**		
1	**Time of transport: Month**	*text, required*	[Min: 1, Max: 12]
2	**Time of transport: Year**	*text, required*	[Min: 2024, Max: 2030]
3	Weekday or weekend day/holiday	*radio button*	1 Weekday<br>2 Weekend day/holiday
4	Departure from collection unit	*time*	[hh:mm]
5	Arrival at destination unit	*time*	[hh:mm]
6	Urgency	*dropdown*	1 Emergency<br>2 < 2 hours<br>3 Disposition during the day<br>4 Elective transport
7	**Location of collection unit**	*text, required*	*text*
8	Medical specialty of collection unit	*dropdown*	1 Pediatrics<br>2 Pediatric surgery<br>3 Internal medicine<br>4 Surgery<br>5 Anesthesiology<br>6 Other
9	Other medical specialty	*text*	*If 'other' was specified*
10	**Collection area**	*dropdown, required*	

			1 Ward (Pediatrics)<br>2 Ward (Adults)<br>3 IMC<br>4 PICU<br>5 NICU<br>6 (Adult-)ICU<br>7 X-ray/endoscopy/CT scanner<br>8 Theatre and recovery<br>9 A & E
11	**Destination unit**	*identifier, required*	*text*
12	**Destination type**	*dropdown, required*	1 PICU<br>2 NICU<br>3 (Adult-)ICU<br>4 IMC<br>5 Ward<br>6 Theatre / cardiac catheter / Emergency intervention<br>7 A & E<br>8 Other
13	Other destination type	*text*	*If 'other' was specified*

**Patient details**			
14	**Is the patient's age less than one year?**	*radio button, required*	1 Yes<br>2 No
15	**patient's age in months**	*text, required*	[Min: 1, Max: 12]
16	**patient's age in years**	*text, required*	[Min: 0, Max: 17]
17	Sex	*dropdown*	1 Male<br>2 Female<br>3 Ambiguous<br>4 Unknown
18	Weight (kg)	*text*	*text*
19	Isolation necessary during transport?	*radio button*	1 Yes<br>2 No
20	Leading symptom necessitating transport	*dropdown*	1 Respiratory<br>2 Cardiac<br>3 Neurologic

			4 Nephrological<br>5 Gastroenterological<br>6 Dermatological<br>7 Musculoskeletal<br>8 Hematopoietic/lymphatic system<br>9 Unknown<br>10 Other
21	Other symptom	*text*	*If 'other' was specified*
22	Cause of leading symptom necessitating transport	*dropdown*	1 Infectious<br>2 Immunological/ rheumatological<br>3 Oncological/hematological<br>4 Transplant (solid organs)<br>5 Transplant (bone marrow)<br>6 Metabolic/endocrinological<br>7 Trauma/surgical<br>8 Burns/scalding/chemical burn<br>9 Unknown<br>10 Other
23	Other cause	*text*	*If 'other' was specified*

**Vitals and blood gas parameters at admission at destination unit**			
24	Mean blood pressure (mmHg)	*text*	*text*
25	SpO_2 (%)	*text*	*text*
26	FiO_2 at time SpO_2 measured (%)	*text*	[Min: 21, Max: 100]
27	Blood gas measured?	*radio button*	1 Yes<br>2 No
28	Blood gas sampling site	*radio button*	1 Arterial<br>2 Capillary<br>3 Venous
29	pH	*text*	[Min: 6.0, Max: 8.0]
30	Base Excess (mmol/l)	*text*	[Min: -40, Max: 40]
31	Lactate (mmol/l)	*text*	[Min: 0, Max: 40]
32	pCO_2	*text*	*text*

33	pCO_2 unit	*radio button*	1 mmHg<br>2 kPa
34	pO_2	*text*	*text*
35	pO_2 unit	*radio button*	1 mmHg<br>2 kPa
36	Subjective severity of illness (documentation of overall impression based on the NACA score)	*radio button*	1 NACA I<br>2 NACA II<br>3 NACA III<br>4 NACA IV<br>5 NACA V<br>6 NACA VI<br>7 NACA VII

**Mode of transport / transport team**			
37	**Mode of transport**	*dropdown, required*	1 Ambulance<br>2 Ambulance with dedicated equipment<br>3 Intensive Care Ambulance<br>4 Air transport<br>5 Intensive Care Air transport<br>6 Other
38	Other mode of transport	*text*	*If 'other' was specified*
39	Additional pediatric equipment brought by the transport team?	*radio button*	1 Yes<br>2 No
40	Type of transport team	*dropdown*	1 Regular crew (non-specialist team)<br>2 Regular crew plus Pediatrics<br>3 PICU team<br>4 NICU team<br>5 Other
41	Other transport team	*text*	*If 'other' was specified*
42	Is a pediatrician (MD1) part of the transport team?	*radio button*	1 Yes<br>2 No

43	Is MD1 team lead?	*radio button*	1 Yes<br>2 No
44	Is MD1 part of the regular crew?	*radio button*	1 Yes<br>2 No
45	Grade of MD1	*radio button*	1 Consultant<br>2 Resident, ≥ 3 years of medical training<br>3 Resident, < 3 years of medical training
46	Specific qualifications of MD1	*radio button (multiple selections possible)*	1 Intensive Care Medicine<br>2 Neonatology<br>3 (Pediatric)Cardiology<br>4 Emergency medicine<br>5 EPALS/PALS<br>6 DIVI Intensive Care Transport Course
47	Is a physician of another specialty a member of the transport team (MD2)?	*radio button*	1 Yes<br>2 No
48	Medical specialty of MD2?	*radio button*	1 Internal medicine<br>2 Anesthesiology<br>3 Surgery<br>4 Other
49	Other medical specialty	*text*	*If 'other' was specified*
50	Is MD2 team lead?	*radio button*	1 Ja<br>2 Nein
51	Is MD2 part of the regular crew?	*radio button*	1 Ja<br>2 Nein
52	Grade of MD2	*radio button*	1 Consultant<br>2 Resident, ≥ 3 years of medical training<br>3 Resident, < 3 years of medical training

53	Specific qualifications of MD2	*radio button (multiple selections possible)*	1 Intensive Care Medicine<br>2 Cardiology<br>3 Emergency medicine<br>4 EPALS/PALS<br>5 DIVI Intensive Care Transport Course
54	Did a critical care nurse accompany the patient?	*radio button*	1 Yes<br>2 No
55	Specific qualification of the nurse	*radio button (multiple selections possible)*	1 Specialist pediatric intensive care/anesthesia nursing<br>2 EPALS/PALS<br>3 DIVI Intensive Care Transport Course
56	Did a perfusionist accompany the patient?	*radio button*	1 Yes<br>2 No
57	Did a parent accompany the patient?	*dropdown*	1 Yes<br>2 No – parent not present<br>3 No – parent declined to accompany<br>4 No – parent not permitted to accompany<br>5 No – parent cannot accom-pany due to space restrictions

**Interventions**			
58	Oxygen	*radio button*	1 By local team before transport<br>2 By transport team<br>3 After transport<br>4 Not done
59	High flow nasal cannula therapy	*radio button*	1 By local team before transport<br>2 By transport team<br>3 After transport<br>4 Not done
60	Non-invasive ventilation	*radio button*	

			1 By local team before transport<br>2 By transport team<br>3 After transport<br>4 Not done
61	Invasive ventilation	*radio button*	1 By local team before transport<br>2 By transport team<br>3 After transport<br>4 Not done
62	Chest drain	*radio button*	1 By local team before transport<br>2 By transport team<br>3 After transport<br>4 Not done
63	NO inhalation	*radio button*	1 By local team before transport<br>2 By transport team<br>3 After transport<br>4 Not done
64	ECMO	*radio button*	1 By local team before transport<br>2 By transport team<br>3 After transport<br>4 Not done
65	Peripheral venous access	*radio button*	1 By local team before transport<br>2 By transport team<br>3 After transport<br>4 Not done
66	Central venous access	*radio button*	1 By local team before transport<br>2 By transport team<br>3 After transport<br>4 Not done
67	Intraosseous access	*radio*	1 By local team before transport<br>2 By transport team<br>3 After transport<br>4 Not done
68	Arterial access	*radio button*	1 By local team before transport<br>2 By transport team<br>3 After transport

			4 Not done
69	Inotrope or vasopressor infusion	*radio button*	1 By local team before transport<br>2 By transport team<br>3 After transport<br>4 Not done
70	Infusion of blood products	*radio button*	1 By local team before transport<br>2 By transport team<br>3 After transport<br>4 Not done
71	Antiarrhythmics	*radio button*	1 By local team before transport<br>2 By transport team<br>3 After transport<br>4 Not done
72	Pacing	*radio button*	1 By local team before transport<br>2 By transport team<br>3 After transport<br>4 Not done
73	Cardioversion/Defibrillation	*radio button*	1 By local team before transport<br>2 By transport team<br>3 After transport<br>4 Not done
74	Analgetics	*radio button*	1 By local team before transport<br>2 By transport team<br>3 After transport<br>4 Not done
75	Sedatives	*radio button*	1 By local team before transport<br>2 By transport team<br>3 After transport<br>4 Not done
76	Muscle relaxants	*radio button*	1 By local team before transport<br>2 By transport team<br>3 After transport<br>4 Not done
77	Antiepileptic drugs	*radio button*	

			1 By local team before transport<br>2 By transport team<br>3 After transport<br>4 Not done
78	ICP monitoring	*radio button*	1 By local team before transport<br>2 By transport team<br>3 After transport<br>4 Not done
79	Urinary catheter	*radio button*	1 By local team before transport<br>2 By transport team<br>3 After transport<br>4 Not done
80	Other drains	*radio button*	1 By local team before transport<br>2 By transport team<br>3 After transport<br>4 Not done
81	CPR	*radio button*	1 By local team before transport<br>2 By transport team<br>3 After transport<br>4 Not done
82	Extracorporeal Life Support (ECLS)	*radio button*	1 By local team before transport<br>2 By transport team<br>3 After transport<br>4 Not done
83	Critical incidents	*radio button (multiple selections possible)*	1 None<br>2 Accidental extubation<br>3 Ventilator failure (ventilator defect, loss of gas supply)<br>4 Loss of all IV access<br>5 Cardiac arrest<br>6 Medication administration error<br>7 Equipment failure or incompatibility<br>8 Other
84	Other critical incidents	*text*	*If 'other' was specified*

Supplementary Table 2: Pädiatrisches Intensivtransport Register (PIT) - Data Dictionary / Item List V1.0 – *German version*

#	Variable	(Text/Button/Dropdown)	Belegung
	**Stammdaten**		
1	**Monat des Transportbeginns**	*Freitext, Pflichtfeld*	[Min: 1, Max: 12]
2	**Jahr des Transportbeginns**	*Freitext, Pflichtfeld*	[Min: 2024, Max: 2030]
3	Wochentag oder Wochenende/Feiertag	*Button*	1 Wochentag<br>2 Wochenende/Feiertag
4	Uhrzeit Transportbeginn	*Uhrzeitfeld*	[Uhrzeit]
5	Uhrzeit Transportende	*Uhrzeitfeld*	[Uhrzeit]
6	Dringlichkeit	*Dropdown*	1 Notfall<br>2 < 2 Stunden<br>3 Im Tagesverlauf disponibel<br>4 disponibel
7	**Name Quellklinik**	*Freitext, Pflichtfeld*	*Freitext*
8	Verlegende Fachrichtung	*Dropdown*	1 Pädiatrie<br>2 Kinderchirurgie<br>3 Innere Medizin<br>4 Chirurgie<br>5 Anästhesie<br>6 Andere
9	Andere Fachrichtung	*Freitext*	*Sofern „andere" angegeben wurde*
10	**Verlegende Station**	*Dropdown, Pflichtfeld*	1 Normalstation (Kinder)<br>2 Normalstation (Erwachsene)<br>3 IMC<br>4 PICU<br>5 NICU<br>6 (Erwachsenen-)ICU<br>7 Bildgebung / Endoskopie<br>8 OP /Aufwachraum<br>9 Notaufnahme / Schockraum
11	**ID Zielklinik**	*Vierstelliger Code, Pflichtfeld, Identifier*	*Freitext*
12	**Zielstation**	*Dropdown, Pflichtfeld*	1 PICU<br>2 NICU<br>3 (Erwachsenen-)ICU

			4 IMC<br>5 Normalstation<br>6 OP/Herzkatheter/ Notfallintervention<br>7 Notaufnahme/Schockraum<br>8 Andere
13	Andere Station	*Freitext*	*Sofern „andere“ angegeben wurde*

**Patientin / Patient**			
14	**Patientenalter < 1 Jahr**	*Button, Pflichtfeld*	1 Ja<br>2 Nein
15	**Alter in Monaten**	*Freitext, Pflichtfeld*	[Min: 1, Max: 12]
16	**Alter in Jahren**	*Freitext, Pflichtfeld*	[Min: 0, Max: 17]
17	Geschlecht	*Dropdown*	1 Männlich<br>2 Weiblich<br>3 Divers<br>4 Unbekannt
18	Patientengewicht (kg)	*Freitext*	*Freitext*
19	Isolationsbedarf während Transport?	*Button*	1 Ja<br>2 Nein
20	Führendes Symptom, das zum Transport geführt hat	*Dropdown*	1 Respiratorisch<br>2 Kardiologisch<br>3 Neurologisch<br>4 Nephrologisch<br>5 Gastroenterologisch<br>6 Dermatologisch<br>7 Muskuloskelettal<br>8 Blutbildendes/lymphatisches System<br>9 Unbekannt<br>10 Sonstige: Freitext
21	Sonstiges Symptom	*Freitext*	*Sofern „sonstige“ angegeben wurde*
22	Ursache für das führende Symptom, das zum Transport geführt hat	*Dropdown*	1 Infektiologisch<br>2 Immunologisch / rheumatologisch<br>3 Onkologisch / hämatologisch<br>4 Transplant (solide Organe)

			5 Transplant (Knochenmark)<br>6 Metabolisch / endokrinologisch<br>7 Traumatologisch / chirurgisch<br>8 Verbrennung / Verbrühung / Verätzung<br>9 Unbekannt<br>10 Sonstige: Freitext
23	Sonstige Diagnose	*Freitext*	*Sofern „sonstige" angegeben wurde*

**Vitalparameter bei Übernahme**			
24	Blutdruck (MAD in mmHg)	*Freitext*	*Freitext*
25	Sauerstoffsättigung (SpO_2 in %)	*Freitext*	*Freitext*
26	FiO_2 zum Zeitpunkt SpO_2-Messung (in %)	*Freitext*	[Min: 21, Max: 100]
27	Blutgasanalyse bei Übernahme durchgeführt	*Button*	1 Ja<br>2 Nein
28	Abnahmeort	*Button*	1 Arteriell<br>2 Kapillär<br>3 Venös
29	pH	*Freitext*	[Min: 6.0, Max: 8.0]
30	Base Excess (in mmol/l)	*Freitext*	[Min: -40, Max: 40]
31	Laktat (in mmol/l)	*Freitext*	[Min: 0, Max: 40]
32	pCO_2	*Freitext*	*Freitext*
33	Einheit pCO_2	*Button*	1 mmHg<br>2 kPa
34	pO_2	*Freitext*	*Freitext*
35	Einheit pO_2	*Button*	1 mmHg<br>2 kPa
36	Subjektive Krankheitsschwere (Dokumentation des Gesamteindrucks anhand des NACA Scores)	*Button*	1 NACA I<br>2 NACA II<br>3 NACA III<br>4 NACA IV<br>5 NACA V<br>6 NACA VI

			7 NACA VII

Transportmittel / -besatzung			
37	**Verwendetes Rettungsmittel**	*Dropdown, Pflichtfeld*	1 RTW<br>2 RTW mit Zusatzausstattung<br>3 ITW<br>4 RTH<br>5 ITH<br>6 Andere
38	Anderes Rettungsmittel	*Freitext*	*Sofern „andere" angegeben wurde*
39	Pädiatrische Zusatzausstattung durch Transportteam mitgebracht?	*Button*	1 Ja<br>2 Nein
40	Art des Transportteams	*Dropdown*	1 Stammbesetzung Rettungsmittel<br>2 Stammbesetzung Rettungsmittel zzgl. Pädiatrie<br>3 Pädiatrisches Intensivteam<br>4 Neonatologisches Intensivteam<br>5 Andere
41	Anderes Transportteam	*Freitext*	*Sofern „andere" angegeben wurde*
42	Pädiater:in Teil des Transportteams? (Ä1)	*Button*	1 Ja<br>2 Nein
43	Teamleitung (Ä1)	*Button*	1 Ja<br>2 Nein
44	Teil der Stammbesetzung (Ä1)	*Button*	1 Ja<br>2 Nein
45	Ausbildungsstand (Ä1)	*Button*	1 Fachärztin/-arzt<br>2 Assistenzärztin/-arzt, ≥ 3 Jahre WB-Zeit<br>3 Assistenzärztin/-arzt, < 3 Jahre WB-Zeit

46	Qualifikation (Ä1)	*Button (Mehrfachauswahl möglich)*	1 Intensivmedizin<br>2 Neonatologie<br>3 (Kinder)Kardiologie<br>4 Notfallmedizin<br>5 EPALS/PALS<br>6 DIVI Intensivtransport
47	Ärztin/Arzt anderer Fachrichtung Teil des Transportteams? (Ä2)	*Button*	1 Ja<br>2 Nein
48	Fachrichtung (Ä2)	*Button*	1 Innere Medizin<br>2 Anästhesie<br>3 Chirurgie<br>4 Andere
49	Andere Fachrichtung	*Freitext*	*Sofern „andere" angegeben wurde*
50	Teamleitung (Ä2)	*Button*	1 Ja<br>2 Nein
51	Teil der Stammbesetzung (Ä2)	*Button*	1 Ja<br>2 Nein
52	Ausbildungsstand (Ä2)	*Button*	1 Fachärztin/-arzt<br>2 Assistenzärztin/-arzt, ≥ 3 Jahre WB-Zeit<br>3 Assistenzärztin/-arzt, ≥ 3 Jahre WB-Zeit
53	Qualifikation (Ä2)	*Button (Mehrfachauswahl möglich)*	1 Intensivmedizin<br>2 Kardiologie<br>3 Notfallmedizin<br>4 EPALS/PALS<br>5 DIVI Intensivtransportkurs
54	Intensivpflege Teil des Transportteams?	*Button*	1 Ja<br>2 Nein
55	Qualifikation Intensivpflege	*Button (Mehrfachauswahl möglich)*	1 Fachpflege pädiatrische Intensiv-/Anästhesiepflege<br>2 EPALS/PALS<br>3 DIVI Intensivtransportkurs

56	Kardiotechniker anwesend?	*Button*	1 Ja<br>2 Nein
57	Elternbegleitung während des Transports (alternativ Bezugsperson)	*Dropdown*	1 Ja<br>2 Nein – nicht vor Ort<br>3 Nein – Eltern wünschen keine Begleitung<br>4 Nein – Eltern dürfen nicht begleiten<br>5 Nein – keine Begleitung aus Platzgründen

**Interventionen**			
58	Sauerstofftherapie	*Button*	1 Vorbestehend<br>2 Durch Transportteam initiiert<br>3 Unmittelbar nach Übergabe<br>4 Nicht durchgeführt
59	HFNC	*Button*	1 Vorbestehend<br>2 Durch Transportteam initiiert<br>3 Unmittelbar nach Übergabe<br>4 Nicht durchgeführt
60	NIV	*Button*	1 Vorbestehend<br>2 Durch Transportteam initiiert<br>3 Unmittelbar nach Übergabe<br>4 Nicht durchgeführt
61	Invasive Beatmung	*Button*	1 Vorbestehend<br>2 Durch Transportteam initiiert<br>3 Unmittelbar nach Übergabe<br>4 Nicht durchgeführt
62	Thoraxdrainage	*Button*	1 Vorbestehend<br>2 Durch Transportteam initiiert<br>3 Unmittelbar nach Übergabe<br>4 Nicht durchgeführt
63	NO-Inhalation	*Button*	1 Vorbestehend<br>2 Durch Transportteam initiiert

			3 Unmittelbar nach Übergabe<br>4 Nicht durchgeführt
64	ECMO	*Button*	1 Vorbestehend<br>2 Durch Transportteam initiiert<br>3 Unmittelbar nach Übergabe<br>4 Nicht durchgeführt
65	PVK	*Button*	1 Vorbestehend<br>2 Durch Transportteam initiiert<br>3 Unmittelbar nach Übergabe<br>4 Nicht durchgeführt
66	ZVK	*Button*	1 Vorbestehend<br>2 Durch Transportteam initiiert<br>3 Unmittelbar nach Übergabe<br>4 Nicht durchgeführt
67	Intraossärer Zugang	*Button*	1 Vorbestehend<br>2 Durch Transportteam initiiert<br>3 Unmittelbar nach Übergabe<br>4 Nicht durchgeführt
68	Arterielle Blutdruckmessung	*Button*	1 Vorbestehend<br>2 Durch Transportteam initiiert<br>3 Unmittelbar nach Übergabe<br>4 Nicht durchgeführt
69	Katecholamine	*Button*	1 Vorbestehend<br>2 Durch Transportteam initiiert<br>3 Unmittelbar nach Übergabe<br>4 Nicht durchgeführt
70	Transfusion von Blutprodukten	*Button*	1 Vorbestehend<br>2 Durch Transportteam initiiert<br>3 Unmittelbar nach Übergabe<br>4 Nicht durchgeführt
71	Antiarrhythmika	*Button*	1 Vorbestehend<br>2 Durch Transportteam initiiert<br>3 Unmittelbar nach Übergabe

			4 Nicht durchgeführt
72	Notfall-Pacer	*Button*	1 Vorbestehend<br>2 Durch Transportteam initiiert<br>3 Unmittelbar nach Übergabe<br>4 Nicht durchgeführt
73	Kardioversion/Defibrillation	*Button*	1 Vorbestehend<br>2 Durch Transportteam initiiert<br>3 Unmittelbar nach Übergabe<br>4 Nicht durchgeführt
74	Analgetika	*Button*	1 Vorbestehend<br>2 Durch Transportteam initiiert<br>3 Unmittelbar nach Übergabe<br>4 Nicht durchgeführt
75	Sedativa	*Button*	1 Vorbestehend<br>2 Durch Transportteam initiiert<br>3 Unmittelbar nach Übergabe<br>4 Nicht durchgeführt
76	Muskelrelaxantien	*Button*	1 Vorbestehend<br>2 Durch Transportteam initiiert<br>3 Unmittelbar nach Übergabe<br>4 Nicht durchgeführt
77	Antiepileptika	*Button*	1 Vorbestehend<br>2 Durch Transportteam initiiert<br>3 Unmittelbar nach Übergabe<br>4 Nicht durchgeführt
78	Hirndrucksonde (EVD/Parenchym)	*Button*	1 Vorbestehend<br>2 Durch Transportteam initiiert<br>3 Unmittelbar nach Übergabe<br>4 Nicht durchgeführt
79	Blasendauerkatheter	*Button*	1 Vorbestehend<br>2 Durch Transportteam initiiert<br>3 Unmittelbar nach Übergabe<br>4 Nicht durchgeführt
80	Andere Drainagen	*Button*	1 Vorbestehend

			2 Durch Transportteam initiiert<br>3 Unmittelbar nach Übergabe<br>4 Nicht durchgeführt
81	Kardiopulmonale Reanimation	*Button*	1 Vorbestehend<br>2 Durch Transportteam initiiert<br>3 Unmittelbar nach Übergabe<br>4 Nicht durchgeführt
82	Extrakorporaler Life Support (ECLS)	*Button*	1 Vorbestehend<br>2 Durch Transportteam initiiert<br>3 Unmittelbar nach Übergabe<br>4 Nicht durchgeführt
83	Gab es auf dem Transport Probleme?	*Button (Mehrfachauswahl möglich)*	1 Keine<br>2 Akzidentielle Extubation<br>3 Versagen der maschinellen Beatmung (Gerätedefekt, Gas leer)<br>4 Verlust des Gefäßzugangs<br>5 Herzkreislaufversagen<br>6 Dosierungsfehler Medikamente<br>7 Gerätefehler / Inkompatibilität<br>8 Andere
84	Andere Probleme	*Freitext*	*Sofern „andere" angegeben wurde*
